# Dietary Nitrate from Beetroot Juice for Hypertension: A Systematic Review

**DOI:** 10.3390/biom8040134

**Published:** 2018-11-02

**Authors:** Diego A. Bonilla Ocampo, Andrés F. Paipilla, Estevan Marín, Salvador Vargas-Molina, Jorge L. Petro, Alexandra Pérez-Idárraga

**Affiliations:** 1 Research Division, DBSS, 110861 Bogotá, Colombia; ndandrespaipilla@gmail.com (A.F.P.); maringarciae@gmail.com (E.M.); salvadorvargasmolina@gmail.com (S.V.); jlpetros@hotmail.com (J.L.P.); info.movenutrition@gmail.com (A.P.-I.); 2 Research Group in Biochemistry and Molecular Biology, Universidad Distrital Francisco José de Caldas, 110311 Bogotá, Colombia; 3 Research Group in Physical Activity, Sports and Health Sciences (GICAFS), Universidad de Córdoba, 230002 Montería, Colombia; 4 Institución Educativa CCAPF, 111511 Bogotá, Colombia; 5 Molecular Biology Laboratory, Dr. Félix Gómez Endocrinometabolic Research Center, University of Zulia, 15424 Maracaibo, Venezuela; 6 EADE-University of Wales Trinity Saint David, 29017 Málaga, Spain; 7 Move Nutrition, 050021 Medellin, Colombia

**Keywords:** *Beta vulgaris*, hypertension, dietary supplements, nitric oxide, blood pressure

## Abstract

According to current therapeutic approaches, a nitrate-dietary supplementation with beetroot juice (BRJ) is postulated as a nutritional strategy that might help to control arterial blood pressure in healthy subjects, pre-hypertensive population, and even patients diagnosed and treated with drugs. In this sense, a systematic review of random clinical trials (RCTs) published from 2008 to 2018 from PubMed/MEDLINE, ScienceDirect, and manual searches was conducted to identify studies examining the relationship between BRJ and blood pressure. The specific inclusion criteria were: (1) RCTs; (2) trials that assessed only the BRJ intake with control group; and (3) trials that reported the effects of this intervention on blood pressure. The search identified 11 studies that met the inclusion criteria. This review was able to demonstrate that BRJ supplementation is a cost-effective strategy that might reduce blood pressure in different populations, probably through the nitrate/nitrite/nitric oxide (NO_3_^−^/NO_2_^−^/NO) pathway and secondary metabolites found in *Beta vulgaris*. This easily found and cheap dietary intervention could significantly decrease the risk of suffering cardiovascular events and, in doing so, would help to diminish the mortality rate associated to this pathology. Hence, BRJ supplementation should be promoted as a key component of a healthy lifestyle to control blood pressure in healthy and hypertensive individuals. However, several factors related to BRJ intake (e.g., gender, secondary metabolites present in *B. vulgaris*, etc.) should be studied more deeply.

## 1. Introduction

Hypertension or high blood pressure (HBP) is a common disease that has become a pandemic for several years. High blood pressure is the main risk factor attributed to many deaths in middle-income countries, and the second, before tobacco, in low and high-income countries. In addition, it is the second risk factor that causes disability-adjusted life years [1]. The number of adults with high blood pressure increased from 594 million in 1975 to 13 billion in 2015, highlighting this increase occurred in low- and middle-income countries [2]. According to the last report of the World Health Organization (WHO), in 2013, HBP was the cause of approximately 45% of deaths from heart disease and 51% of deaths from stroke, which represents a total of 9.4 million deaths per year [3].

It is widely known that some factors related to diets, such as excessive sodium intake, high consumption of alcoholic drinks, low intake of fruits and vegetables, and a sedentary lifestyle, could increase the prevalence of HBP. It has also been stated that deficiency of some vitamins, such as folic acid, riboflavin, and vitamins C and D, can be considered risk factors to develop this non-communicable disease [4]. Faced with this situation, scientific organizations, such as the American Heart Association (AHA), have recommended dietary approaches to stop hypertension (DASH), alongside the Mediterranean diet, as effective nutritional strategies included in the treatment of HBP [5]. The best-proven nonpharmacological interventions for the prevention and treatment of HBP, especially by means of the reduction of arterial systolic blood pressure (SBP), include weight loss, healthy diet, reduced intake of dietary sodium, enhanced intake of dietary potassium, physical activity, and moderation in alcohol intake. During normotension, these inventions are able to reduce between 2 and 4 mmHg SBP, while in HBP there is a reduction between 4 and 11 mmHg SPB [6]. 

Taking into consideration the fact that HBP appears to have a complex association with endothelial dysfunction, a phenotypical alteration of the vascular endothelium, which precedes the development of cardiovascular events, could result in future cardiovascular risk; therefore, it is essential to achieve action regarding the above factors [7]. Nitric oxide (NO), a molecule that is usually synthesized in the endothelium, could have a substantial effect on the maintenance of vascular homeostasis, either by its potent dilator effect, systemic blood pressure control, or atherogenesis delay [8]. Recently, many studies have focused their attention on the positive effects of some functional foods, e.g., beetroot juice (BRJ). In fact, BRJ serves as a strategy that could not only increase exercise performance (see Reference [9] for a review), but also favor the blood pressure parameters control in healthy subjects and hypertensive patients (in any of their categories with or without pharmacological treatment), possibly through a higher synthesis of NO.

**Figure 1:**
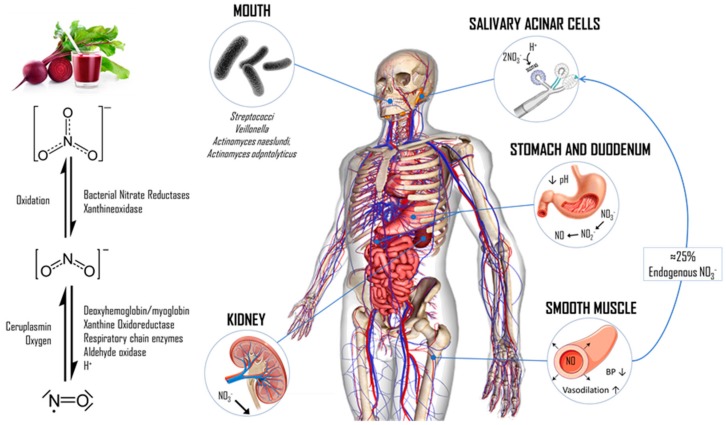
The nitrate/nitrite/nitric oxide (NO_3_^−^/NO_2_^−^/NO) pathway after beetroot juice (BRJ) ingestion. Next to BRJ ingestion, oral microbiota on the posterior surface of the tongue is able to reduce NO_3_^−^ to NO_2_^−^ by means of their enzymatic machinery. The strict anaerobes *Veillonella atypical* and *Veillonella dispar* are the most important NO_3_^−^ reducers; however, *Actinomyces*, *Rothia*, *Prevotella*, *Neisseria*, and *Haermophilus* are also present on the oral cavity. Even though this non-enzymatic reduction process continues in stomach, where more NO_2_^−^ and NO are produced due to the acid environment, a considerable amount of NO_3_^−^ from blood (≈25%) is taken up by an electrogenic 2NO_3_^−^/H^+^ symporter called SLC17A5 (also known as sialin, UniProt ID: Q9NRA2) in the salivary gland acinar cells [14]. Both dietary and saliva NO_3_^−^, and its reduced forms NO_2_^−^ and NO, enter directly to systemic circulation after the absorption process in the stomach and intestine. Thus, the increase of NO_3_^−^ and NO_2_^−^ concentrations in blood allow the generation of NO by either enzymatic or non-enzymatic mechanisms (such as xanthine oxidoreductase, respiratory chain enzymes, aldehyde oxidase, methemoglobin formation, protons, etc.), especially under physiologic hypoxia and low pH [12]. Because of its short half-life (1–2 ms), once NO is produced in blood it is broken down by hemoglobin or it can diffuse into the vascular smooth muscle cells and binds to guanylyl cyclase, which allows the allosteric activation of this last and subsequent cGMP production. Here, cGMP acts as a second messenger and activates PKG, which in turn can modulate smooth muscle relaxation by several interlinked mechanisms: (i) activation of K^+^ channels leading to hyperpolarization; (ii) reduction of intracellular Ca^2+^ concentration; and (iii) activation of the myosin-light-chain phosphatase [15]. Finally, NO_3_^−^ is normally excreted in the urine by the kidneys. BP: blood pressure. Original material.

In brief, a significant proportion of nitrate (NO_3_^−^) is present in BRJ (≈25%) as well as in some other vegetables like spinach, rocket, cress, lettuce, celery, and radish (>250 mg NO_3_^−^/100 g), which concentrates in saliva and comes into contact with symbiotic bacteria on the dorsal surface of the tongue that reduce inorganic NO_3_^−^ to nitrite (NO_2_^−^) through bacterial nitrate reductases (i.e., xanthine oxidase). This saliva rich in nitrogen compounds reaches the stomach where a small part of the NO_2_^−^ is reduced to NO through a non-enzymatic reaction, which is favored by the acidic environment of this organ. However, most of the NO_3_^−^ and NO_2_^−^ are quickly absorbed by the stomach and duodenum to get into systemic circulation [10]. Interestingly, 20–25% of NO_3_^−^ is reabsorbed from the bloodstream and concentrated in the salivary glands to later be a substrate of the bacteria as mentioned above and produce NO_2_^−^ that is swallowed again for its subsequent reduction [11]. This generates a significant increase in the concentration of these ions in the plasma (up to 182 ± 55 µM after 1–2 h equivalent to ≈550% increase for NO_3_^−^ and 373 ± 211 nM after 2–3 h equivalent to ≈400% increase for NO_2_^−^), which favors the production of NO in the wall of blood vessels and erythrocytes by employing reduction mechanisms of an enzymatic nature (e.g., xanthine oxidoreductase, respiratory chain enzymes, and aldehyde oxidase), and non-enzymatic (e.g., deoxygenated hemoglobin/myoglobin, protons, vitamin C, and polyphenols). Nonetheless, this reduction process is stimulated during conditions with low oxygen availability and an acidic pH, which allows the synthesis of NO to be localized at certain specific times [12]. In this way, the increase in NO concentration promotes vasodilation through different cellular mechanisms (e.g., cyclic guanosine monophosphate (cGMP)/cGMP–dependent protein kinase (PKG) pathway and hyperpolarization/relaxation after activation of K^+^ channels), and it is associated with a significant decrease in blood pressure to muscle relaxation in the endothelium [13]. A summary of this process is outlined in Figure 1.

Even when the production of NO decreases with age, and this could be associated with increased risk of hypertension and cardiovascular disease in the elderly, recent meta-analyzes [10,16] have shown the positive effects of NO_3_^−^ dietary intake on blood pressure. To our knowledge, this is the first time that a systematic review of the evidence from randomized controlled trials (RCTs) investigating the effect of BRJ on SBP and diastolic blood pressure (DBP) is conducted, since previous reviews mixed dietary nitrate sources.

## 2. Materials and Methods 

The present systematic review was conducted according to established guidelines, and it is reported according to the preferred reporting items for systematic reviews and meta-analyses (PRISMA) guidelines [17]. It was also submitted to PROSPERO, an international database of prospectively registered systematic reviews developed by the University of York, NY, USA (https://www.crd.york.ac.uk/prospero; CRD42018112041).

### 2.1. Search Strategy and Data Sources

The RCT searching was carried out through the databases PubMed/MEDLINE and Science Direct, and further papers were sought by hand-searching. The data search was performed by using free language terms related to BRJ and blood pressure. The search string for all databases was the following “*beetroot juice*” *OR* “*red beet*” *OR* “*beta vulgaris*” *AND (blood pressure or hypertension)*. This search was enriched with terms *NOT exercise NOT sport*. The data search was performed during June 2018.

### 2.2. Eligibility Criteria and Data Extraction

Specific inclusion criteria were as follows: (1) studies written in English; (2) published from 2008 onwards, as this would cover the most recent years of research; (3) RCTs; (4) trials that only evaluated the consumption of BRJ by using a control group, regardless of the gender; and (5) trials that reported the effects of these interventions on blood pressure. On the other hand, exclusion criteria were: (1) studies that did not correspond to original research (e.g., editorials, notes, reviews, etc.); (2) studies where their object of study was the effects of BRJ on exercise or sports performance; (3) studies that did not assess the effects on blood pressure; (4) studies that used NO_3_^−^ salts as a dietary supplement; and (5) studies with no control group.

After the search of the published articles, the filters options of the databases were used to meet the inclusion criteria 1 to 3. The remaining references were filtered by screening the title, abstract, or full text publication. The study selection, risk of bias, and data extraction was performed independently by two of the authors (AFP and DAB). Risk of bias of all included RCTs was assessed using the Cochrane risk of bias tool [18]: selection bias, performance bias, detection bias, attrition bias, reporting bias, and any other bias. Discrepancies were identified and resolved through discussion (with a third author where necessary). The primary outcome was considered to be changes on systolic and diastolic blood pressure. Selected publications met all the inclusion criteria and went on to the next phase of data analysis and synthesis, which is explained in the next subsection.

### 2.3. Data Synthesis

The following data were obtained and analyzed from the selected studies: (1) characterization of the study population; (2) study length; (3) BRJ dosage; (4) NO_3_^−^ content; (5) placebo; (6) effect on systolic BP; and (7) effect on diastolic BP. All randomized participants in the analysis were included, as it was the least biased way to analyze intervention effects. 

## 3. Results

**Figure 2:**
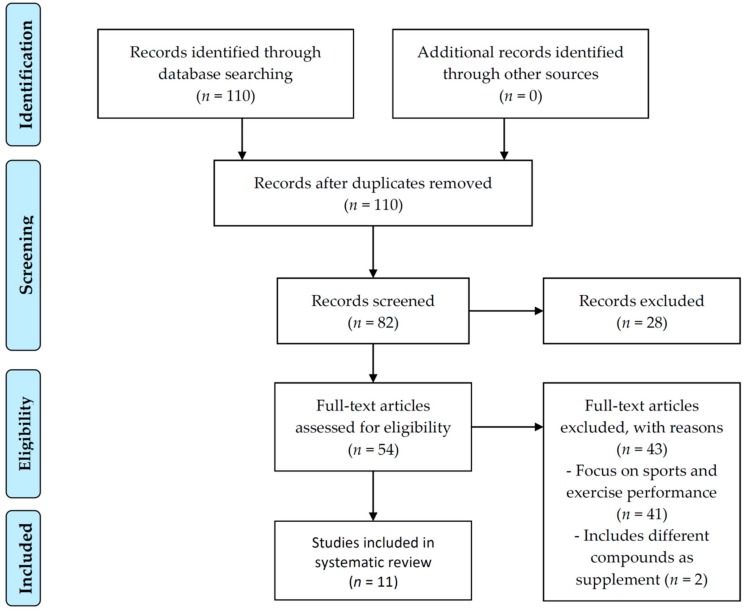
Preferred reporting items for systematic reviews and meta-analyses (PRISMA) flow chart.

The literature selection after using the search terms and Boolean operators resulted in 110 references. A screening of articles after filtration by species, publication date, article type, and text availability resulted in 54 potentially eligible studies. However, after checking the full texts of these studies, 41 of them were excluded since they were focused on exercise/sports performance and two because they included inorganic NO_3_^−^ as dietary supplement. A total of 11 studies met the inclusion criteria. A flow chart of the literature search is shown in Figure 2.

**Figure 3:**
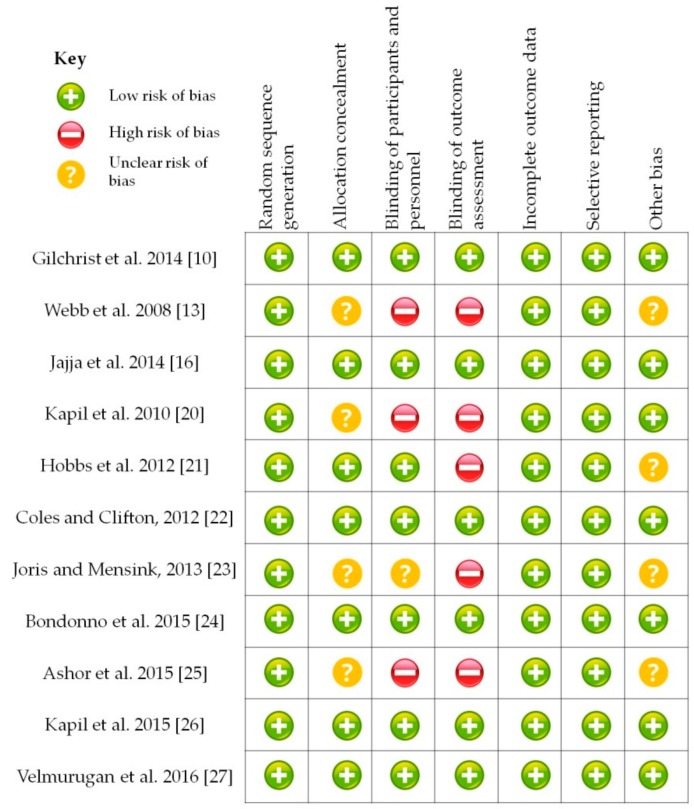
Risk of bias summary.

Results from a total of 310 participants who were represented across the reviewed studies showed there is evidence suggesting that dietary supplementation with BRJ has a positive effect in reducing blood pressure, mainly on SBP compared to DBP. Nonetheless, on certain factors that probably modify this response are the characteristics of the intervened subjects themselves (age, gender, nutritional status, and baseline blood pressure), and the type of intervention performed in the supplementation protocol (duration, BRJ volume, and NO_3_^−^ concentration) (Table 1). The methodological quality of the trials is summarized in Figure 3. Thus, in the following sections, these heterogeneous results will be discussed to provide practical recommendations regarding the BRJ consumption as a possible strategy for the prevention and treatment of high blood pressure.

## 4. Discussion

### 4.1. Effects on Intervention Measures

To analyze the clinical relevance in reducing blood pressure as a result of BRJ consumption, it is important to note that a decrease between 5 and 12 mmHg of SBP and between 5 and 6 mmHg of DBP is associated with a 14–38% risk reduction in stroke, from 9 to 16% risk reduction of mortality from coronary heart disease, 21% risk reduction of mortality due to coronary disease, and 7% risk reduction in mortality from all causes [27]. It is important to note that, after consumption of BRJ, there were no side effects or adverse interactions reported in the subjects (medicated or not) included in the studies of this systematic review; in fact, Kapil et al. 2015 [25] suggested a role for dietary NO_3_^−^ as an adjuvant therapy.

#### 4.1.1. Age

Most of the studies reviewed found positive effects after supplementation with BRJ in healthy adult subjects [13,19,20,21,22,23]. Notwithstanding, the reviewed studies that have included older adults are scarcer and usually have a lower response to supplementation [10,22,23], although some of the publications report a decrease in blood pressure after BRJ consumption in these subjects [16,24,25,26]. This variation in the results may be due to the fact that aging is related to a lower sensitivity of the vascular components to the beneficial effects of NO_3_^−^ coming from the diet, probably due to a lower rate of non-enzymatic conversion of NO_3_^−^/NO_2_^−^ to NO, also to a reduction in the response of the endothelium and vascular smooth muscle cells to NO. Alike, it has been made evident that aging produces changes in the oral microbiota and the gastric acid production, which could negatively influence the efficiency of the conversion of NO_2_^−^ into NO. Despite the aforementioned, Bahadoran et al. [28] reported in a recent meta-analysis that there is a positive relation between age and the effect of BRJ supplementation on SBP. Therefore, more research is needed to clarify this age-dependent variation after supplementation with dietary NO_3_^−^. 

#### 4.1.2. Gender

Regarding gender, most of the study subjects were men. Few studies have evaluated whether there is a difference in blood pressure values between women and men after dietary supplementation with NO_3_^−^ using BRJ. Kapil et al. [19] reported that there was a greater response to supplementation of SBP values in men compared to women, which is probably because during the pre-menopausal period they tend to register lower values of baseline blood pressure, such as those reported. In the study, higher plasma levels of NO_2_^−^ could limit the reduction of blood pressure values after dietary intervention. In the same way, Cole and Clifon [21] observed a stronger trend in the decrease of SBP in men than in women supplemented with BRJ, to the point that when analyzing the groups separately at 6 h post-consumption, reductions in SBP of 4–5 mmHg and 2–3 mmHg were reported for men and women, respectively. It is not clear if this phenomenon was the result of sexual characteristics per se or if, perhaps, as the authors describe, the age difference between both genders (36.2 ± 2.9 in men and 48.9 ± 3.1 in women) could have influenced the variables analyzed. Nowadays, the body of evidence does not allow us to conclude differences by gender, which means that more research is needed to clarify the response of SBP and DBP values after supplementation with BRJ in men and women.

#### 4.1.3. Nutritional Status

Studies like that of Jajja et al. [16] and Ashor et al. [29] where patients with some degree of obesity or being overweight obtained positive effects on their blood pressure with BRJ intervention. In this way, BRJ supplementation had a greater effect on SBP compared to DBP, which agrees with the research of Bahadoran et al. [28], who found in their meta-analysis that overweight subjects experienced a greater reduction in SBP than subjects of normal weight (11.3 mmHg compared to 6.0 mm Hg, respectively). It could be taken as a preventive strategy to reduce the risk of cardiovascular diseases for this type of population that is more predisposed and at risk of cardiovascular events [30].

#### 4.1.4. Baseline Blood Pressure

Currently, there is some degree of controversy regarding whether supplementation with BRJ benefits hypertensive patients receiving pharmacological treatment or healthy subjects with slightly elevated blood pressure values. In this study, we found seven studies showing a greater effect in healthy subjects and four studies that find positive effects in patients with hypertension.

**Figure 4:**
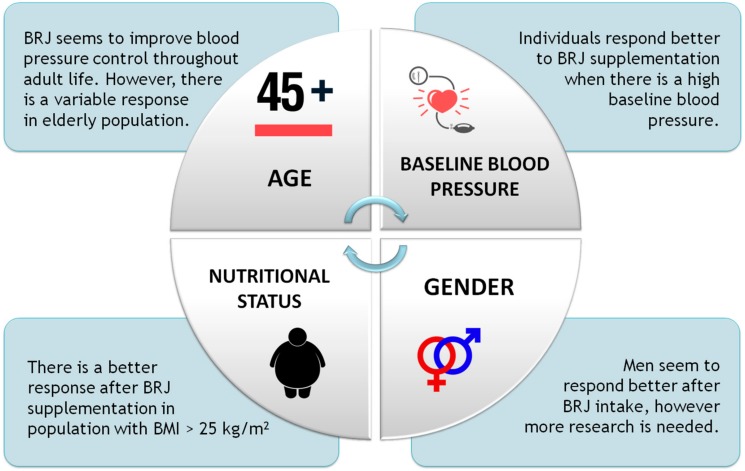
Individual factors influencing the effects of BRJ supplementation on blood pressure. Dietary administration of BRJ has been associated with beneficial effects on SBP and DBP; however, these effects appear to depend on age, gender, baseline blood pressure, body weight, and body composition.

On the one hand some studies, such as Bondonno et al. [23], have not found a significant effect in reducing blood pressure after BRJ supplementation during a week in hypertensive patients with medical treatment. It has been hypothesized that the use of drugs can affect the production of nitric oxide, besides the fact that is more difficult to obtain an additional benefit when considering subjects with controlled blood pressure values. On the other hand, Kapil et al. [25] found a significant reduction in patients with hypertension under pharmacological treatment after a four-week intervention with BRJ. In this study, the magnitude of the decrease in blood pressure was equivalent to what would be achieved after treatment with an anti-hypertensive drug, so we can infer that when starting from a high baseline blood pressure, supplementation with BRJ could have greater effects on the stabilization of blood pressure values. In fact, this has recently been confirmed by Bahadoran et al. [28], who conclude that those individuals with high SBP values present a more significant decrease in the levels of this variable after supplementation with BRJ. No significant changes have been seen in DBP [28]. See Figure 4 for a schematic overview. 

### 4.2. Factors Related to Beetroot Juice Administration 

#### 4.2.1. Nitrate Concentration

During supplementation with BRJ, the amount of NO_3_^−^ for the studies included in this systematic review was between 300 and 500 mg NO_3_^−^ (equivalent to ≈5–8 mmol NO_3_^−^), which is higher than the acceptable daily intake of NO_3_^−^ as defined by the WHO (3.7 mg/kg body weight per day) [31]. It has been established that a positive correlation between the concentration of inorganic NO_3_^−^ and the hypotensive effect [32]. However, nowadays there is some discussion regarding whether the hypotensive effect is actually due to the concentration of NO_3_^−^ or whether other components of the BRJ mediate this physiological response, such as betalains, oxalic acid, hydroxycinnamic acids, among others. In this sense, some studies have not shown significant changes on blood pressure after the administration of NO_3_^−^-depleted BRJ versus NO_3_^−^-rich BRJ [10,22,23]. Furthermore, Bahadoran et al. [28] reported in their recent meta-analysis a weak effect size of trials that used NO_3_^−^-depleted BRJ as a placebo on blood pressure values. This generates the need to evaluate the role of NO_3_^−^-depleted BRJ and its effect on blood pressure and other health markers, bearing in mind that future research could consider the effect of other compounds present in BRJ, such as betalains.

It is important to highlight that large variations (from 0.01 to 2.4 g/L) in the NO_3_^−^ content of commercial BRJ have been found previously, where the variety Mona Lisa might be the optimal recommended source of beetroot raw material, due to the high NO_3_^−^ concentration (4.6 g/L) [33]. It becomes clear that more research is needed to establish differences between varieties among countries.

#### 4.2.2. Volume

The BRJ volumes reported in the studies of this review had a reasonably wide range, ranging from 70 to 500 mL. In general, there was no significant difference between the volumes administered; however, the recent meta-analysis by Bahadoran et al. [28] suggested that there is a more significant effect on blood pressure when about ≈500 mL of the supplement is supplied, making it clear that this work analyzed the effects of BRJ and highlight its potential NO_3_^−^-independent effects.

#### 4.2.3. Length

From the experimental design of the 11 revised RCTs, there are acute and chronic supplementation protocols, where the blood pressure analysis was carried out between 24 h and six weeks after BRJ administration. There are better effects at the postprandial level during the first 3 h and up to 24 h after the consumption of BRJ. Additionally, when evaluating the effect of beet supplementation, it is concluded that interventions over two weeks generated better results [16,24,26], compared to those with a duration of one week [23] (Table 1).

## 5. Conclusions

In conclusion, BRJ supplementation might be an easy, accessible, safe, and evidence-based strategy to reduce blood pressure. Its attractive cost-effectiveness ratio would benefit pre-hypertensive patients when pharmacological treatment should not be the first alternative. The potential reduction in blood pressure after BRJ administration might contribute to the diminishment in mortality rate for cerebrovascular diseases [34]. This systematic review showed that BRJ supplementation has a great potential to reduce the SBP and DPB values in both healthy subjects and those with cardiovascular risk (pre- and hypertensive patients). The most probable mechanism is the NO_3_^−^/NO_2_^−^/NO pathway, although more research is required to establish if other secondary metabolites of BRJ may mediate the effect (e.g., betalains). Individual factors influencing the effects of BRJ supplementation on blood pressure encompass baseline blood pressure, overweight/obese status, gender, and age. It is recommended that an administration period of minimum two weeks is used in order to have sustained results; however, more research is needed to evaluate the relevance and long-term effect of BRJ administration in hypertensive individuals. This reduction in blood pressure, especially SBP, not only would decrease morbidity and mortality, but it would also decrease public health expenditure.

## Figures and Tables

**Table 1:** Evidence for the effects of beetroot juice (BRJ) supplementation on blood pressure (BP). Table summarizing the main results of eleven studies included in the systematic review.

Reference	Sample Population and Gender	Age and BMI	Baseline Blood Pressure (SBP; DBP)	Supplementation Duration	BRJ Dosage	NO_3_^−^ Concentration	NO_3_^—^Depleted as Placebo?	Effect on SBP	Effect on DBP
Gilchrist et al. 2014 [10]	27 both	67.2 ± 4.9 years	142.9 ± 13.9;	14 days	250 mL	30.7 mM;	Yes	NS	NS
	(18 M; 9 F)	30.8 ± 3.2 kg/m^2^	81.1 ± 9.2			7.6 mmol			
Webb et al. 2008 [13]	14 both	25.5 ± 4.5 years	108.0 ± 1.3;	Acute	500 mL	45.0 ± 2.6 mM;	No	↓ 10.4 ± 3.0 mmHg after 2.5 h	↓ 8.1± 2.1 mmHg after 3 h
						22.5 mmol;			
	(9 M; 5 F)	22.54 kg/m^2^	70.3 ± 1.0			2.79 g/L			
Jajja et al. 2014 [16]	21 both	62.0 ± 1.4 years	129.8 ± 19.1;	21 days	70 mL	≈69.1–92.1 mM;	Yes	↓ 7.3 ± 5.9 mmHg during final week	NS
	(12 M; 9 F)	30.1 ± 1.2 kg/m^2^	77.1 ± 15.4			≈4.8–6.4 mmol;			
						300–400 mg			
Kapil et al. 2010 [19]	9 both	18–45 years	120.6 ± 4.1;	Acute	250 mL	22.4 ± 3.8 mM;	No	↓5.4 ± 1.5 mmHg after 3 h	NS
		18–40 kg/m^2^	70.9 ± 2.5			5.6 mmol			
Hobbs et al. 2012 [20]	18 M	31.4 ± 3.0 years	130.6 ± 3.2;	Acute with different dosages	500 mL	4.6, 11.4, and 22.8 mM; 2.3, 5.7, and 11.4 mmol	No	↓ 13.1, 20.5, and 22.2 mmHg according to [NO_3_^−^] after 2–3 h	↓ 16.6, 14.6 y 18.3 mmHg according to [NO_3_^−^] after 2–3 h
		24,4 ± 3.0 kg/m^2^	82.1 ± 5.6						
Coles and Clifton, 2012 [21]	30 both	42.5 ± 3.4 years	132.4 ± 1.6;	Acute	500 g	15 mM;	No	↓ 4–5 mmHg after 6 h only in men	NS
	(15 M; 15 F)	28.2 ± 1.3 kg/m^2^	81.1 ± 1.2			7.5 mmol			
Joris and Mensink, 2013 [22]	20 M	61 ± 7 years	135.2 ± 18.2;	Acute	140 mL	57.59 mM; 8.06 mmol; 500 mg	Yes	NS	↓ 3–6 mmHg after 1–4 h
		30.1 ± 1.9 kg/m^2^	93.2 ± 12.0						
Bondonno et al. 2015 [23]	27 both	63.2 ± 4.4 years	132.9 ± 11.8;	7 days	140 mL	49.99 mM; 6.99 mmol; 3.1 g/L	Yes	NS	NS
	(10 M; 17 F)	26.9 ± 3.2 kg/m^2^	76.2 ± 10.4						
Ashor et al. 2015 [24]	21 both	62.0 ± 4.5 years	135.1 ± 14.9;	21 days	70 mL	≈69.1–92.1 mM;	Yes	↓ 10 mmHg after 3 weeks	↓ 3 mmHg after 3 weeks
	(12 M; 9 F)	29.9 ± 4.2 kg/m^2^	77.5 ± 9.6			≈4.8–6.4 mmol;			
						300–400 mg			
Kapil et al., 2015 [25]	32 both	56.3 ± 16.4 years	138.4 ± 17.1;	4 weeks	250 mL	25.7 ± 5.3 mM; 6.4 mmol	Yes	↓ 7.7 mmHg after 24 h and 4 weeks	↓ 5.2 and 2.4 mmHg after 24 h and 4 weeks
	(16 M; 16 F)	26.5 ± 4.0 kg/m^2^	82.8 ± 11.2						
Velmurugan et al., 2016 [26]	33 both	53.3 ± 10.1 years	125.2 ± 15.1;	6 weeks	250 mL	24.2 ± 7.7 mM; 6.05 mmol	Yes	↓ 4.1 mmHg after 6 weeks	↓ 1.5 mmHg after 6 weeks
	(12 M; 21 F)	26.8 ± 4.9 kg/m^2^	76.3 ± 8.6						

M: Male; F: Female; BMI: Body mass image; BRJ: Beetroot juice; SBP: Systolic blood pressure; DBP: Diastolic blood pressure; NO_3_^–^: Nitrate; NS: No statistically significant changes.
